# High yield of protein-rich forage rice achieved by soil amendment with composted sewage sludge and topdressing with treated wastewater

**DOI:** 10.1038/s41598-020-67233-w

**Published:** 2020-06-23

**Authors:** Luc Duc Phung, Megumi Ichikawa, Dung Viet Pham, Atsushi Sasaki, Toru Watanabe

**Affiliations:** 1 The United Graduate School of Agricultural Sciences, Iwate University, 3-18-8 Ueda, Morioka, Iwate, 020-8550 Japan; 2 Faculty of Agriculture, Yamagata University, 1-23 Wakaba-machi, Tsuruoka, Yamagata, 997-8555 Japan; 3 Faculty of Engineering, Yamagata University, 4-3-16 Jonan, Yonezawa, Yamagata, 992-8510 Japan

**Keywords:** Agroecology, Wetlands ecology

## Abstract

Aiming to promote low-cost production of protein-rich forage rice and resource recycling from wastewater treatment plants, a pot experiment was conducted to assess the possibility to substitute mineral fertilizers with composted sewage sludge (CSS) with/without top-dressing with treated municipal wastewater (TWW). Results indicated that a basal application of CSS at 2.6 g N pot^−1^ replaced conventional mineral fertilization of 1.3 g N pot^−1^ to produce comparable yields with the same rice protein content, although there might be a risk of increased As concentration in rice grains. Interestingly, CSS application at a reasonable dose of 1.3 g N pot^−1^, followed by a topdressing with TWW resulted in 27% higher yield and 25% superior rice protein content relative to the mineral fertilization, with no risk of heavy metal(loid) accumulation in grains and in paddy soils. Here we demonstrated an appealing fertilization practice with zero use of mineral fertilizers in paddy rice cultivation, expectedly contributing towards sustainable rice farming and animal husbandry in Japan.

## Introduction

Accelerated generation of sewage sludge and treated municipal wastewater (TWW) along with increasing limitations in availability of resources have required highly efficient and sustainable disposal practices for the sludge and TWW, triggering a rapid change of wastewater treatment plants (WWTPs) from stations for wastewater purification and pollutant removal towards facilities for recourse recovery^1^. Rice farming demands large quantities of irrigation water and mineral fertilizers, which allows the great opportunity for recycling plant nutrients and water from WWTPs to reduce the dependence of the crop production on mineral fertilizers and other freshwater sources through soil amendment with sewage sludge and reuse of TWW.

Aiming to substitute forage rice for imported feed-stuffs of unstable supply and a price highly dependent on global markets and to avoid the risk of rising production costs of domestic animal husbandry, adoption of forage rice cultivation is drawing increasing attention in Japan^2,3^. In attempts to promote low-cost forage rice cultivation while recycling plant nutrients and water from WWTPs, we have developed new irrigation systems for effective reuse of TWW as a sole source of both water and plant nutrients to produce high grain yields of protein-rich rice without the need for mineral fertilizers^3–5^. These TWW irrigation systems have proved highly beneficial and applicable to paddy fields located near local WWTPs, from which the effluent is supplied to paddy fields^3^; however, they frequently face difficulties to attain an even distribution of TWW to paddy fields far from the WWTPs due to the rising cost of instalment of water-distributing pipes/channels. Hence, application of sewage sludge might be an alternative in those paddy fields owing to its abundance in organic matter and plant nutrients, especially N and P^6^. Nevertheless, soil amendment of sewage sludge generally raises concerns of environmental and health risks due to its contained contaminants, for example, hazardous heavy metals^7^. Thus, in order to make the most of valuable plant nutrients while minimizing the risk of hazardous pollution, suitable methods of sewage sludge application should be considered.

Composting sewage sludge is an effective way to enhance its quality and suitability for agricultural use by limiting the availability of heavy metal(loid)s to plants, as well as eliminating pathogens from the composted sewage sludge (CSS)^8^. Effects of CSS on important crops such as maize, wheat, barley, and some vegetables have been widely investigated^9–15^; however, studies on the effects of CSS on rice (*Oryza sativa* L.), especially forage rice, are surprisingly scarce. Most studies have evaluated the influence of soil amendment with either raw sewage sludge or sewage sludge biochar instead of CSS on rice growth and paddy soils, in which a substantial improvement of soil fertility and considerable increases in rice yield upon application of sewage sludge have been demonstrated^16–21^, which might eliminate or at least reduce the need for exogenous mineral fertilizers.

We hypothesized that CSS can replace mineral fertilizers. Therefore, this study aimed to evaluate the feasibility of using CSS as total or partial substitute for conventional mineral fertilization while targeting a high yield of protein-rich forage rice. Since TWW also contains substantial amounts of organic and inorganic nutrients beneficial for plant growth and development, combining soil amendment with CSS as basal application and topdressing with TWW might be an appealing strategy for fertilization management in paddy fields, which has not been studied to date. Therefore, here, we evaluated effects of such management practices on rice growth and development, rice yield and nutrition quality, soil fertility, and accumulation of heavy metal(loid)s in rice grains and in paddy soils.

## Results

### Growth parameters

Plant height of rice plants examined in this experiment responded differently to the fertilization treatments (*p* < 0.05). Relative to the control that was supplemented with mineral fertilizers (T1, Table 1), most of the CSS-applied treatments could maintain comparable plant heights (*p* > 0.05), except T4, which received a single CSS fertilization at the same total N input as the control, significantly lowered the height of rice plants (*p* < 0.05).

**Table 1:** Description of N doses and fertilization practices examined in the study.

Treatment	Basal application		Topdressing		Total N rate (g pot^−1^)
	Source	N rate (g pot^−1^)	Source	N rate (g pot^−1^)	
T1 (Control)	MF	0.8	MF	0.5	1.3
T2	CSS	0.8	MF	0.5	1.3
T3	CSS	0.8	TWW	1.5	2.3
T4	CSS	1.3	—	0	1.3
T5	CSS	1.3	TWW	1.8	3.1
T6	CSS	2.6	—	0	2.6

MF: mineral fertilizer, CSS: composted sewage sludge, TWW: treated municipal wastewater.

Tillering capacity of the rice plants was significantly influenced by the examined treatments (*p* < 0.05). Compared with the control (42.3 tillers pot^−1^), T2 and T3 that replaced the mineral fertilizers in basal application with CSS at the same dose of 0.8 g N pot^−1^ (Table 1) lessened tiller number by 16 and 19%, respectively, although topdressing with either mineral fertilizers (T2) or TWW (T3) was done. The same trend was seen in T4, in which the mineral fertilizers for both basal and topdressing were completely substituted with a single application of CSS at 1.3 g N pot^−1^. However, basal fertilization with CSS at 1.3 g N pot^−1^ and topdressing with TWW (T5) could produce similar tiller number (42 tillers pot^−1^) as the control. A sole application of CSS at 2.6 g N pot^−1^ yielded the highest tiller number of 48.3 tillers pot^−1^.

Results of shoot dry mass shows similar trend with tiller number of the rice plants as affected by the examined fertilization practices (Table 2). T2, T3 and T4 had significantly lower shoot dry weights (59.2, 67.7, and 55.0 g pot^−1^, respectively) while T5 and T6 produced comparable shoot biomass (76.1 and 81.5 g pot^−1^, respectively) relative to T1 (80.2 g pot^−1^).

**Table 2:** Growth parameters of rice plants as affected by the examined treatments.

Treatment	SPAD value		Plant height (cm)	Tiller number (tillers pot^−1^)	Shoot mass (g pot^−1^)
	Tillering	Flowering			
T1 (control)	48.4 ± 1.2	37.5 ± 2.1^bc^	94.7 ± 2.3^ab^	42.3 ± 3.5^ab^	80.2 ± 4.2^a^
T2	48.3 ± 0.2	36.2 ± 1.5^c^	99.3 ± 4.7^a^	35.3 ± 4.0^b^	59.2 ± 3.9^cd^
T3	48.2 ± 0.9	44.5 ± 1.4^a^	91.7 ± 2.9^abc^	34.3 ± 2.5^b^	67.7 ± 4.4^bc^
T4	49.7 ± 0.5	34.0 ± 0.0^c^	84.0 ± 1.7^c^	38.3 ± 4.0^b^	55.0 ± 3.7^d^
T5	49.6 ± 0.9	45.3 ± 1.2^a^	92.7 ± 3.1^ab^	42.0 ± 1.0^ab^	76.1 ± 4.3^ab^
T6	50.1 ± 0.3	40.0 ± 1.0^b^	87.7 ± 1.5^bc^	48.3 ± 4.2^a^	81.5 ± 4.7^a^

Data are means ± standard deviation of three replications. Different letters in a row indicate significant difference (*p* < 0.05), whereas similar letters and no letter indicate no significant difference among treatments.

**Figure 1:**
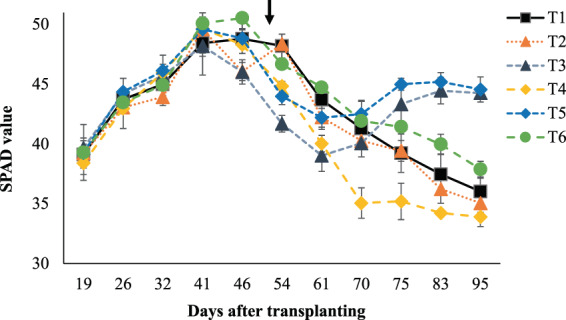
SPAD values of rice leaves during the tillering and flowering stages. Error bars indicate standard deviations of three replications. The arrow denotes the time of topdressing.

The examined fertilization practices had no effect on leaf chlorophyll content expressed as SPAD values of rice leaves during the tillering stage (*p* > 0.05), whereas the rice plants had variety in SPAD records during the flowering time (*p* < 0.05) (Table 2). From 75 days after transplanting (DAT) onwards, SPAD values were within the same range between the control and T2, T4, and T5, while only T3 and T5 that received topdressing with TWW had significantly higher SPAD records (Table 2, Fig. 1).

### Grain yield and rice nutritional quality

**Figure 2:**
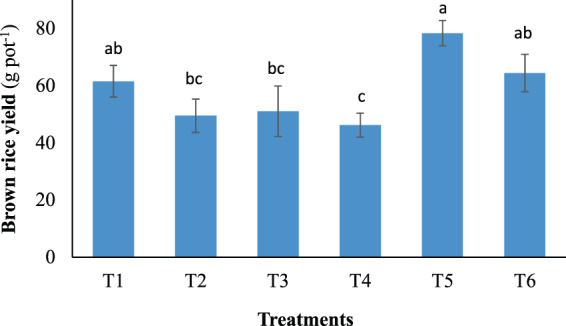
Brown rice yield as affected by the different fertilization treatments. Error bars indicate standard deviations of three replications. Data with unshared letters are significantly different (*p* < 0.05).

Grain yields of the rice plants were presented here as the yields of brown rice, and responded differently (*p* < 0.05) to the fertilization treatments (Fig. 2). Compared with the control (61.6 g pot^−1^), T2, T3, and T4 notably reduced the yield by approximately 20, 17, and 25% respectively. However, T5 and T6 produced higher yields of 27 and 5% relative to the control, respectively. Interestingly, at the same amount of CSS amendment (1.3 g N pot^−1^), T4 without topdressing resulted in the lowest yield (46.2 g pot^−1^) while T5 receiving topdressing with TWW produced the highest grain weight (78.4 g pot^−1^) among the examined treatments (Fig. 2).

**Figure 3:**
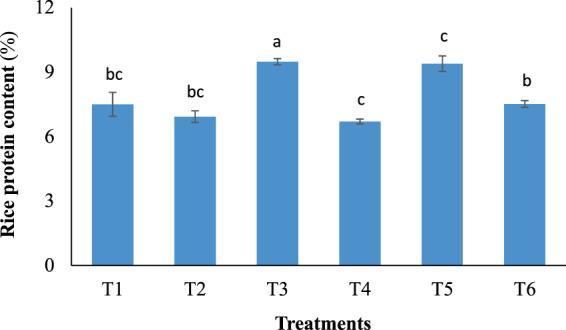
Rice protein content as affected by the different fertilizer treatments. Error bars indicate standard deviations of three replications. Data with unshared letters are significantly different (*p* < 0.05).

Nutritional quality of the forage rice is evaluated based on rice protein content, which responded differently to the examined fertilization treatments (Fig. 3). Although T2 and T4 received the same total N input as the control (1.3 g N pot^−1^), their brown rice had 8–11% lower protein contents compared with that of the control. T6 with the double N dose of 2.6 g pot^−1^ yielded a comparable rice protein content (7.5%) as the control. Interestingly, only treatments that received topdressing with TWW (T3 and T5) showed significantly higher protein levels (9.5 and 9.4%) compared with the control.

### Heavy metal(loid) concentrations in rice grains

Concentrations of five examined elements including Zn, Cu, Pb, Cd, and As are shown in Table 3. Concentration of Zn in the brown rice harvested from all CSS-amended treatments was similar or lower than that recorded from the control, ranging between 0.5 and 0.9 mg kg^−1^, although the differences were statistically non-significant (*p* > 0.05). In contrast, there was a significant difference in Cu concentration, in which case the highest concentration was observed in the control (6.1 mg kg^−1^), followed by T2, T4, and T6 (5.0, 4.8, and 4.4 mg kg^−1^, respectively). The lowest Cu concentration (3.9 mg kg^−1^) was found in the brown rice produced under T3 and T5. Generally, CSS application either as a single amendment (T4 and T6) or in combination with topdressing by TWW irrigation (T3 and T5) or mineral fertilizers (T2) did not increase concentrations of these two micro-nutrients in the brown rice.

**Table 3:** Concentration of heavy metal(loid)s in the brown rice.

Treatment	Examined element (mg kg^−1^)				
	Zn	Cu	Pb	Cd	As
T1	0.9 ± 0.6	6.1 ± 0.5^a^	0.05 ± 0.03	0.01 ± 0.0066	0.23 ± 0.006^c^
T2	0.8 ± 0.2	5.0 ± 0.6^ab^	0.04 ± 0.03	0.01 ± 0.0003	0.26 ± 0.012^bc^
T3	0.6 ± 0.1	3.9 ± 0.3^b^	0.06 ± 0.04	ND	0.27 ± 0.056^bc^
T4	0.9 ± 0.1	4.8 ± 0.3^ab^	0.08 ± 0.06	ND	0.34 ± 0.007^a^
T5	0.5 ± 0.1	3.9 ± 0.2^b^	0.08 ± 0.04	ND	0.23 ± 0.012^c^
T6	0.7 ± 0.2	4.4 ± 1.1^b^	0.08 ± 0.07	ND	0.30 ± 0.025^ab^
ML	NA	NA	0.2	0.4	0.35
JPS	NA	NA	3	1	2

Data are means ± standard deviation of three replications. Different letters in a row indicate significant difference (*p* < 0.05), whereas similar letters and no letter indicate no significant difference among treatments. ML: maximum levels for contaminants and toxin in food^22^, JPS: Japanese standard for animal feed^23^, ND: not detected, NA: not available.

Replacing mineral fertilizers in the control with CSS at 0.08 g N pot^−1^ (T2 and T3) didn’t increase concentration of Pb in the brown rice, although the higher CSS applications of 1.3 and 2.6 g N pot^−1^ (T4, T5, and T6) likely showed a higher accumulation levels of the element. However, these differences were non-significant (*p* > 0.05, Table 3). Concentrations of Cd in the brown rice of all treatments were negligible. In case of As, most of the CSS treated rice plants yielded higher As concentrations in their rice grains compared with that of the control, except T5 that showed a similar level of As accumulation in the rice grain (Table 3). Overall, despite remarkable differences in absorption of Pb, Cd, and As among the treatments, contents of these elements were under the limits set by FAO/WHO^22^ and the standards for animal feed in Japan^23^.

### Plant nutrients and heavy metal(loid) concentrations in paddy soil

**Figure 4:**
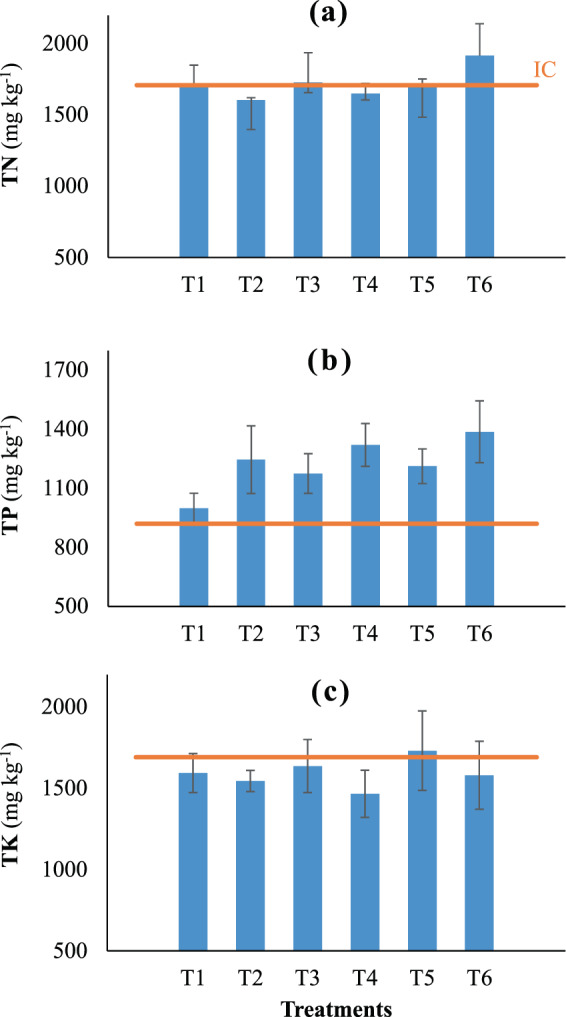
Nutrition of the postharvest soils. Error bars indicate standard deviations of three replications. IC: initial contents of the elements in the soil before the experiment.

Figure 4 shows the contents of three essential macro-nutrients (N, P, and K) in paddy soil before and after the experiment. Although there was no significant difference in total N, P and K (TN, TP, and TK, respectively) among the soils fertilized separately with the treatments tested herein (*p* > 0.05), there were still remarkable variation among them. Generally, concentration of TN in the soil was relatively stable despite marked differences in total N input among the treatments (Table 1). Most treatments maintained soil TN levels comparable or close to the initial TN concentration of the soil before the crop season, except for a slight increase of 12% observed in T6 (Fig. 4a). As for P, all treatments increased soil TP, with CSS-amended treatments (T2, T3, T4, T5, and T6) showing notably higher TP contents than the control (Fig. 4b). In contrast, most soils experienced a decrease in K after harvesting, except for slight increments in T5 (Fig. 4c), which received the moderate dose of CSS along with TWW irrigation (Table 1).

The effect of the fertilization treatments tested herein on the accumulation of heavy metal(loid)s in paddy soils is shown in Table 4. Compared with the control, concentrations of Zn and Cu in the soils amended with CSS increased notably (*p* < 0.05), except the case of T2 that maintained concentrations of Zn and Cu at similar levels. All the soils amended with CSS (T2, T3, T4, T5, and T6) tended to have lower Pb concentrations relative to the soils supplied with conventional mineral fertilizers. Further, Cd likely increased due to CSS application, while As concentration did not show a clear trend. Compared with concentrations of these elements in the soil before the experiment, CSS addition tended to increase Zn and Cu contents while slightly reduced Pb concentrations. In turn, the concentration of Cd in the CSS-added soils remained unchanged, compared with the initial soil, while there was a significant reduction in As in the paddy soils after the crop season, regardless of fertilization treatments.

**Table 4:** Concentration of heavy metal(loid)s in paddy soils as affected by the examined fertilization treatments.

Treatment	Examined element (mg kg^−1^)				
	Zn	Cu	Pb	Cd	As
T1	53.6 ± 1.8^bc^	10.6 ± 0.7^ab^	13.2 ± 0.7^a^	0.1 ± 0.0	2.9 ± 0.4^ab^
T2	53.2 ± 1.6^c^	10.3 ± 0.8^b^	11.8 ± 0.4^b^	0.1 ± 0.0	3.0 ± 0.1^ab^
T3	59.2 ± 2.8^ab^	11.3 ± 1.0^ab^	11.7 ± 0.2^b^	0.2 ± 0.0	3.2 ± 0.1^a^
T4	61.4 ± 4.3^a^	13.1 ± 1.9^ab^	12.7 ± 0.7^ab^	0.2 ± 0.0	3.0 ± 0.2^ab^
T5	58.3 ± 1.5^abc^	10.9 ± 0.6^ab^	12.9 ± 0.2^ab^	0.2 ± 0.0	2.8 ± 0.1^ab^
T6	61.1 ± 1.7^a^	14.7 ± 2.8^a^	12.9 ± 0.5^ab^	0.2 ± 0.0	2.5 ± 0.3^b^
Initial soil	60.7 ± 5.4	10 ± 0.3	13.6 ± 0.7	0.2 ± 0.0	6.3 ± 0.1
MPL	300	100	100	3	20

Data are means ± standard deviation of three replications. Data with unshared letters in the same column are significantly different (Tukey’s HSD, α = 0.05). MPL: maximum permissible limits in agricultural soil^33^.

## Discussion

Sufficient fertilization is an essential requirement for vigour growth and development of rice plants^24^. Being the most yield-limiting nutrient in rice production, N is generally applied through high doses of mineral fertilizers to ensure high levels of rice production, especially for high-yielding cultivars such as the forage rice ‘Bekoaoba’ used in this study^25,26^. In this experiment, we have substituted the mineral fertilizers in the control partly or totally with different N doses via CSS application with/without topdressing with TWW (Table 1).

The positive influence of fertilization generally results in effective photosynthesis activities expressed as leaf greenness or SPAD values that strongly associate with N status in rice leaves^5,24,27^. Similar SPAD values among the examined treatments during the tillering stage (before topdressing time, Fig. 1) suggests that the rice plants can acquire nutrients, especially N, from CSS as effectively as from the mineral fertilizers, to support their normal N assimilation during the first growth period. However, the N assimilation is not always along with some other growth parameters. In particular, the lowest plant height recorded in T4 reveals that a single application of CSS at 1.3 g N pot^−1^ to compensate for the same N amount supplied by the mineral fertilizers in the control is likely not effective to sustain the vital growth and development of the rice plants for a long term, though plant height is not a growth trait directly affect the final grain production^24^. On the other hands, tillering is highly important for rice yield due to its relationship with panicle formation^24,27^. Variations in the rice tillering capacity under the different examined treatments were certainly owing to the differences in N rates, since N is the most important nutrient affecting tillering capacity in rice^27^. Overall, among the alternative treatments amended with CSS, only T5 and T6 could produce a comparable and higher tiller number relative to the control, likely because they had received 2.4 and 3-fold higher total N inputs than the control, respectively. This also probably explains for the comparable shoot dry mass recorded in T5 and T6 relative to the control. In short, the most favorable fertilization to ensure vigor growth and development of the rice plants should be either a single application of CSS at a high dose of 2.6 g N pot^−1^, or CSS amendment at a more reasonable rate of 1.3 g N pot^−1^ combined with TWW topdressing, which can additionally provide the rice plants substantial amount of N due to the high concentration of N (24.8 mg L^−1^) in the irrigation TWW.

Generally, high shoot dry mass in rice is important because it is closely associated with high grain yield as a result of the assimilate transportation from rice shoots to panicles^24,27^. Higher yielding rice cultivars were reported to have close relation with higher dry matter production^27^. Thus, the similar shoot dry mass retained in T5 and T6 relative to the control also explains for the comparable yields they produced as the control did. The highest yield observed in T5 was likely due to not only the highest total N input, but additionally, to the combination of CSS (1.3 g N pot^−1^) with TWW irrigation, which could supply the highest N rate for topdressing (1.8 g pot^−1^, Table 1) among all treatments. Significantly higher rice protein content archived in T3 and T5 compared with other treatments was attributed to topdressing with TWW during the late growing period, regardless of the CSS application rates. The high N concentration (24.8 mg L^−1^) contained in the irrigation TWW could substantially increase the total N inputs in T3 and T5 up to 2.3 and 3.1 g pot^−1^, respectively (Table 1). This might also further explain for the higher shoot dry mass recorded in T3 and T5 compared with that in T2 and T4, respectively, which received the same CSS doses for the basal application (Table 1). During panicle formation, available soil N can become deficient, whereby topdressing is generally carried out at this time to support reproductive growth stage. Topdressing with TWW continuously supplied N during the grain filling stage (from 60 DAT onwards), which helped maintain leaf greenness at high levels (Fig. 1), and subsequently produce more carbohydrates for grain filling. The lowest rice protein content among all treatments observed in T4 was likely owing to the low carbohydrate metabolism during the reproductive stage due to the lack of topdressing fertilization. Previously, strong effect of late N topdressing on grain protein accumulation was reported^28^, in which treatments receiving late N topdressing yielded 30–60% higher rice protein content than treatments without N application during the grain filling stage. Similarly, N application in late growth periods after heading was also demonstrated to improve both N accumulation in panicles and grain yield^29,30^. Relative to our target to achieve high yield of protein-rich rice, topdressing with TWW appeared to be highly feasible following basal soil amendment with CSS. Overall, conventional fertilization with mineral fertilizers (1.3 g N pot^−1^) can be substituted by soil amended with CSS at 1.3 g N pot^−1^ in combination with TWW topdressing (T5), which not only produced higher grain yield but superior rice protein content as well.

The results of heavy metal(loid) accumulation in the brown rice was likely not associated with CSS application and TWW irrigation. Previously, a slight accumulation of Zn and Cu was demonstrated in a perennial ryegrass grown in CSS-amended soils^31^. The same trend was observed in *Mangifera persicforma* trees applied with CSS that promoted the plant growth and increased the accumulation of both elements in roots, stems, leaves and whole plants^12^. Other researches have also demonstrated an increase in heavy metal concentrations in above ground parts of rice with application of sewage sludge. Particularly, it was found that a 10- and a 7-fold increase in Pb and Cd content, respectively, in rice grain were recorded under the effects of co-application of sewage sludge and mineral fertilizers^18,32^. However, in the present study, there was no notable adverse effect of CSS application as well as TWW topdressing on accumulation of the examined elements in the rice grains. Although minor variation in the concentrations of the examined elements was observed among the CSS-amended treatments, and between those and the control, all the concentrations were lower than the maximum limits recommended by FAO/WHO^22^ and the Japanese standard^23^ (Table 1), suggesting that the harvested rice grains are safe to use as feedstuffs. Nevertheless, it is noteworthy that the high doses of CSS in T4 and T6 might raise a concern about accelerated accumulation of As in the rice grains, as their As contents (0.34 and 0.3 mg kg^−1^, respectively) were fairly close to the maximum (0.35 mg kg^−1^) level allowed for food (Table 3). Thus, further monitoring accumulation of As in rice grains produced as staple food from rice fields amended with CSS are essential for ensuring the safety of the rice for human consumption.

The measurement of plant nutrients in the soil after harvesting revealed that CSS could sustain the soil fertility without the use of mineral fertilizers. This was supported by similar levels of TN observed in all treatments, regardless of the fertilization methods. The increment of TP in the soil amended with CSS was likely attributed to the high concentration of P_2_O_5_ in CSS (3.5%), as shown by the increasing trend of soil TP with increasing doses of CSS applied (Fig. 4b). Higher concentration of TK in T5 might be owing to the relatively high concentration of K (10.2 mg L^−1^) in the TWW used for irrigation. Overall, all the alternative fertilization practices tested out in this study can maintain or even improve paddy soil nutrition as effectively as mineral fertilizers. The slight increase in concentrations of Zn, Cu and Cd in the paddy soil after the crop season is not surprising, given that the concentration of these metals in CSS (340, 330, and 0.6 mg kg^−1^, respectively) is higher than that commonly measured in conventional paddy soils, whereas CSS application and TWW topdressing did not caused any clear changes in concentrations of Pb and As in the paddy soils. Importantly, the concentrations of the examined elements were below the maximum permissible limits recommended by the World Health Organization^33^. Nevertheless, long term measurement of these elements in paddy soils applied with CSS and topdressing with TWW should be carried out to assess the build-up possibility of these elements in the soils.

In conclusion, under this laboratory experiment, CSS has been evidenced to be capable to substitute for mineral fertilizers as long as suitable doses and/or topdressing practices are established. High yields of rice with preferable protein contents could be achieved when conventional mineral fertilizers (1.3 g N pot^−1^) were replaced by a single CSS application at the double rate of 2.6 g N pot^−1^ (T6) or at a more reasonable CSS rate (1.3 g N pot^−1^) in combination with TWW irrigation as topdressing (T5). Especially, since T5 showed synergistic effects on rice production, as it not only increased grain yield significantly but additionally resulted in higher protein content, such combination of the reasonable CSS rate as basal and TWW irrigation as topdressing seems one of the most appealing fertilization practices to reduce the dependence of rice cultivation on mineral fertilizers while promoting recycling of plant nutrients from WWTPs, which is likely capable to reduce the amount of plant nutrients discharged into surface water bodies from paddy fields and from WWTPs. Given that these findings were under the lab-scale demonstration; we strongly suggest to carry out follow-up field experiments to ensure the reliability of the favorable fertilization practices figured out herein. Nevertheless, the outcome of this study can serve as a starting point to seek sustainable alternatives to the use of mineral fertilizers in paddy rice cultivation to reduce the production cost of forage rice, which might ensure the sustainability of rice farming and domestic animal husbandry in Japan. Future researches under real field conditions over a long period are also suggested to thoroughly evaluate the potential risks of heavy metal(loid) accumulation in the rice-soil systems.

## Methods

### Experimental materials

A pot experiment was carried out in an open-side greenhouse at the Faculty of Agriculture, Yamagata University in Tsuruoka City, Japan. Wagner pots with an area of 0.05 m^2^ were filled with 7 kg of sandy loam soil collected from experimental plots in the faculty paddy fields. The chemical properties of the soil were as follows: pH 5.5; total C, N, P, and K contents were 19510, 1710, 921, and 1839 mg kg^−1^, respectively.

The CSS used in this experiment was provided by a local WWTP. Concentration of N, P_2_O_5_, and K_2_O in the CSS were 2.5, 3.5, and 0.4%, respectively; water content was 35% and C/N was 9. Concentrations of Zn, Cu, Pb, Cd, and As in the CSS were 340, 330, 9.4, 0.6, and 1.8 mg kg^−1^, respectively.

The TWW used for topdressing was collected from the effluent of the WWTP, which employed the standard activated sludge process followed by chlorine disinfection, showing annual average total suspended solids and biochemical oxygen demand of 3.1 and 4.7 mg L^−1^, respectively^34^. Other basic properties of the TWW are as follows: pH of 7.2; EC of 64.8 mS m^−1^, total N, P, and K contents of 24.8, 0.6, and 10.2 mg L^−1^, respectively.

Uniform 30-day-old seedlings of a forage rice (*Oryza sativa* L., cv. Bekoaoba) were selected for transplanting on May 18^th^, 2018.

### Experimental design

The experiment was laid out in a completely randomized design with six treatments and three replications for a total of 18 experimental pots. Since N is generally the most important limiting nutrient for rice yield^35^, our study focused on evaluating different doses of N as basal and topdressing fertilization as shown in Table 1.

The experiment included one control treatment (T1) comprising the application of mineral fertilizers at a rate of 0.8 g N pot^−1^ (N:P_2_O_5_:K_2_O = 14:14:14) as basal, and at 0.5 g N pot^−1^ (N:K_2_O = 20:20) as topdressing. These were equivalent to 160 kg N-P_2_O_5_-K_2_O and 100 kg N-K_2_O ha^−1^, respectively. For T2, the mineral fertilizer used as basal application was substituted by CSS at 0.8 g N pot^−1^ (equivalent 160 kg N ha^−1^) while the topdressing remained unchanged compared with the control. T3 was the same as T2 except that the topdressing was replaced by TWW irrigation applied as fertigation. T4 and T6 consisted in CSS amendment at 1.3 and 2.6 g N pot^−1^, equivalent to 260 and 520 kg N ha^−1^, respectively, as basal fertilization without topdressing. T5 was amended with CSS at a rate of 1.3 g pot^-1^ as basal application and complemented with TWW irrigation as topdressing fertigation.

The basal fertilization either by application of mineral fertilizers or soil amendment with CSS was conducted one day before transplanting, and topdressing was carried out at 50 DAT. Irrigation with TWW in T3 and T5 was performed by watering the pots with TWW every day from 50 DAT until harvesting to compensate for water loss due to evapotranspiration; other than that, tap-water was daily added to all pots to maintain the water level of 5 cm above the soil surface.

## Data collection

Rice growth parameters including plant height and tiller number were monitored at the maximum tillering stage of the rice plants. Leaf greenness was measured weekly from heathy top leaves of each plants using a SPAD chlorophyll meter (SPAD-502 Chlorophyll Meter, Minolta Co. Ltd., Japan). At harvest, shoot dry mass (g) was measured as the weight of rice shoot oven-dried at 80 °C for 48 h. Grain yield were assessed as the weight of brown rice and was adjusted to 15% moisture content. Nutritional quality of the harvested grains was evaluated based on their protein content. Briefly, the protein content was calculated by multiplying a conversion factor of 5.95^36^ by total N content of brown rice determined by an automatic high-sensitivity NC analyser (Sumigraph NC-220F, SCAS, Japan). Bioaccumulation of the heavy metal(loid)s (Zn, Cu, Pb, Cd, and As) in the rice grains and their concentrations in paddy soils were determined by the standard wet-digestion method followed by measurement with either an inductively coupled plasma mass spectrometer (ICP-MS) (Elan DRC II, PerkinElmer, Japan) for Zn, Cu and Pb or by atomic absorption spectrometry (AAS Model AA7000 equipped with hydride generator, Shimadzu Corporation, Japan) for As^37^. Changes in N, P, and K contents in the paddy soil were also investigated following standard methods as previous studies^3–5^.

### Statistical analysis

Statistical analysis was performed using software package SPSS 24.0 (IBM Corp. Released 2016. IBM SPSS Statistics for Windows, Version 24.0. Armonk, NY: IBM Corp.). Data were subjected to one-way analysis of variance (ANOVA) to test the significance of treatment effects on the examined parameters. Means of significant treatment effects were separated by Tukey’s honestly significant difference (HSD) at *p* < 0.05 probability level.
